# Microstructural Changes in *Vanilla planifolia* Beans after Using High-Hydrostatic-Pressure Treatment in the Curing Process

**DOI:** 10.3390/foods13020177

**Published:** 2024-01-05

**Authors:** Katia D. Rivero-Angeles, Génesis V. Buitimea-Cantúa, Gloria Dávila-Ortiz, Edgar O. López-Villegas, Jorge Welti-Chanes, Zamantha Escobedo-Avellaneda, Darío I. Téllez-Medina

**Affiliations:** 1 Departamento de Ingeniería Bioquímica, Escuela Nacional de Ciencias Biológicas, Instituto Politécnico Nacional, Wilfrido Massieu 399, Gustavo A. Madero, Ciudad de México 07738, Mexico; kriveroa2000@alumno.ipn.mx (K.D.R.-A.); gdavilao@ipn.mx (G.D.-O.); 2 Escuela de Ingeniería y Ciencias, Tecnologico de Monterrey, Av. Eugenio Garza Sada 2501 Sur, Monterrey 64849, Mexico; genesis.vidal@tec.mx (G.V.B.-C.); jwelti@tec.mx (J.W.-C.); 3 Central de Instrumentación de Microscopía, Escuela Nacional de Ciencias Biológicas, Instituto Politécnico Nacional, Prolongación de Carpio y Plan de Ayala S/N, Casco de Santo Tomás, Azcapotzalco, Ciudad de México 11340, Mexico; eolopez@ipn.mx

**Keywords:** *Vanilla planifolia*, curing, high hydrostatic pressure (HHP), microscopy, microstructure

## Abstract

During vanilla bean curing, the cell arrangement derived from the killing technique applied to start bean ripening is essential to obtain the characteristic aroma and flavor of vanilla. Hence, killing is an important step to release the enzymes and compounds required for vanillin production. In this work, high hydrostatic pressure (HHP) at 100–400 MPa for 5 min, using water at 7 °C as the pressure-transmitting medium, was applied as the killing method, and its effect on the microstructural changes in vanilla beans during different curing cycles (C_0_–C_20_) was evaluated and compared with that observed after scalding by using water at 100 °C for 8 s. Microstructural changes in the cross-sectioned beans were analyzed using a stereomicroscope (SM), confocal laser scanning microscopy (CLSM), and environmental scanning electron microscopy (ESEM). The vanilla beans were cross-sectioned and three main sectors were analyzed: the total, annular, and core. The morphometric descriptors, namely, area, Feret’s diameter, and circularity, were quantified via digital image analysis (DIA), from which a shrinkage ratio was calculated. The results show that the total area in the beans presented a maximum decrease in the C_16_ of curing. The core area was most affected by the HHP treatment, mainly at 400 MPa, rather than scalding. CSLM observations revealed the autofluorescence of the compounds inside the beans. In conclusion, the use of microscopy techniques and DIA allowed us to determine the microstructural changes in the HHP-treated pods, which were found to be more numerous than those found in the scalded beans.

## 1. Introduction

*Vanilla planifolia* is an orchid native to the tropical forests of eastern Mexico [1,2]. According to the FAO, in 2021, Mexico was positioned in fourth place of worldwide vanilla production with 609.57 tons, after Madagascar (3070.63 tons), Indonesia (1456.09 tons), and China (877.10 tons) [3]. The orchid is used to obtain a flavor extract with applications in the food, beverage, cosmetic, tobacco, and pharmaceutical industries [4,5]. This flavor extract represents 3–5% of the total bean’s mass, while the remaining mass (approximately 95%) is usually discarded. Proximate analysis of vanilla residues has reported its main composition to be sugars and fiber [6,7]. Vanilla is the second most expensive spice in the world after saffron. The high value of vanilla is because pollination must be performed manually, after being harvested, and because of the long curing process required for flavor development. During curing, the compounds responsible for vanilla’s flavor are synthesized. The traditional curing method is characterized by four phases: killing, sweating, slow drying, and conditioning [8]. Killing methods traditionally consist of vanilla pod immersion in hot water, which aims to start the bean ripening stage by disrupting its cell arrangement. Sweating and drying are performed simultaneously in multiple sweating–drying cycles until reaching a low moisture content (approximately 25%, under sunlight during mornings and under shadow during afternoons, at >60% environmental humidity) [7]. During the killing, the first and most important stage, vegetative growth is inhibited, and the cell tissue structure is destroyed. The latter is necessary to enable the interaction between the hydrolytic enzymes and phenolic precursors required for flavor development [9]. Immersion in hot water or scalding is a common method of pod killing; however, it has been observed that killing does not cause great changes in the microstructure of the beans [8]. Thus, the application of other technologies to facilitate cellular decompartmentalization is of utmost importance.

Applications of high hydrostatic pressure (HHP) technology have attracted the interest of food manufacturers since the process improves the extraction of bioactive compounds and simultaneously involves microbial destruction at very low or moderate temperatures, which allows the preservation of bioactive nutrients and a decrease in food allergenicity [10,11,12]. In addition, it has been shown that, under certain treatment conditions, HHP generates microstructural changes in plant tissues. Yi et al. [13] observed alterations in cell morphology in asparagus treated with HHP at 400 and 600 MPa (for 20 min and 30 min, respectively, at 20 °C), finding extensive disruption and disorganization in cellular structures. Treatments at 100–300 MPa/2 min/20 °C were reported to cause firmness losses in carrots [14], while 400 MPa/30 min/5 °C caused structural changes in cauliflower and spinach [15]. In persimmon fruit, the application of HHP caused cell wall disruption and intracellular dispersion throughout the tissue, together with some nutritionally interesting compounds (tannins, fibers, and carotenoids) [16]. It has been observed that along vanilla beans, there is a heterogeneous distribution of these compounds; β-glucosidase activity, which catalyzes the chemical bond cleavage between phenolic compounds and sugar molecules, is distributed in between the placenta, mesocarp, and trichomes in a 7:2:1 proportion [17]. As for glucovanillin, it is mainly stored in the placenta (92%), with a small proportion in the trichomes (7%) [18]. The use of HHP during the killing stage, hence, would cause microstructural changes to facilitate the enzyme–substrate interaction in vanilla pods. These microstructural changes in vanilla pods have not been reported hitherto, nor compared to those observed after traditional killing techniques are applied. Digital image analysis (DIA) and microscopy techniques used together are powerful tools for understanding microstructural changes in vegetal tissues, as shown by Morales-Delgado et al. [19] and Sánchez-Segura et al. [20] in strawberry and *Capsicum* tissues, respectively.

The purpose of the present study was to evaluate the microstructural changes in vanilla beans during the HHP-assisted curing process compared with scalding through microscopical techniques. Stereoscopic microscopy (SM), confocal laser scanning microscopy (CLSM), environmental scanning electron microscopy (ESEM), and DIA were applied in a quantitative and qualitative evaluation of the vanilla beans.

## 2. Materials and Methods

### 2.1. Plant Material

About 10 kg of mature green vanilla beans (*Vanilla planifolia*) was harvested in Papantla, Veracruz, Mexico, in 2019.

### 2.2. HHP Treatment of Vanilla Beans and Curing Process

The mature green vanilla beans were prepared as described by Buitimea-Cantúa et al. [9], washed with tap water, and organized into groups of 50 beans for each treatment. As a control treatment, each group of 50 green beans was scalded by immersion in 4 L of tap boiling (94 °C) water for 8 s. For HHP treatment, the samples were placed in 11 × 8 cm^2^ polyethylene bags, vacuum-sealed, and treated in a Hiperbaric H135 unit (Burgos, Spain) at 100, 200, 300, and 400 MPa for 5 min, using water at 7 °C as the pressure transmitting medium [21].

After pressure treatment, the vanilla beans were cured for 20 cycles in an incubator (POL-EKO KK400 TOP+/FIT P, Wodzisław Śląski, Poland) under controlled temperature, relative humidity (RH), and light conditions. Each cycle lasted 24 h and consisted of sweating (45 °C/>90% RH) and drying stages (50 °C/60% RH/light) to simulate the curing conditions traditionally used in situ (in Veracruz). Once the 20 drying–sweating cycles were complete, the vanilla beans were conditioned within cellophane bags in an incubator at 25 °C and 50% RH for 30 days. Throughout this vanilla curing process, beans were sampled as follows: cycle 0 (C_0_), cycle 1 (C_1_), cycle 3 (C_3_), cycle 5 (C_5_), cycle 7 (C_7_), cycle 10 (C_10_), cycle 14 (C_14_), cycle 16 (C_16_), cycle 20 (C_20_); additionally, green beans (GB) were used as reference [21].

### 2.3. Microstructural Analysis

The vanilla beans sampled in the curing cycles for each killing treatment were transversally cut using a microtome (Leica, Deer Park. IL, USA) in portions or slices of 3 mm thickness. The cuts were performed in a zone of the vanilla beans near to the pseudo-prismatic region (half-length of the beans). These bean slices were placed into a desiccator for 24 h at room temperature and afterwards stored within 11 × 8 cm^2^ polyethylene bags and sealed under vacuum (EVD4, Torrey, Guadalupe, Nuevo Léon, México) until analysis.

#### 2.3.1. Stereomicroscopy (SM)

From the bean slices mentioned above, images were captured using a stereomicroscope (Stemi SV 11, Carl Zeiss, Thüringen, Germany) with a total magnification of 8× and using reflected light for a bright field. Images were obtained from 4 different slices of each of the applied killing treatments [22].

#### 2.3.2. Confocal Laser Scanning Microscopy (CLSM)

Each bean slice was mounted on glass slides and observed via CLSM (Carl Zeiss, LSM800, Oberkochen, Germany), using the lambda operation mode, which consists of capturing an image sequence at different wavelengths to detect autofluorescence.

Identification of spectral channels was performed using Zeiss efficient navigation (ZEN) software, version 2.6 (blue edition). RGB color fields were acquired, with a 20× and 40× apochromatic objectives using 0.8 and 1.3 numerical apertures, respectively, all of which were stored in TIFF format with a resolution of 2048 × 2048 pixels [6].

#### 2.3.3. Environmental Scanning Electron Microscopy (ESEM)

The slices of vanilla beans were placed on aluminum stubs with double-sided carbon conductive tape and observed under an environmental scanning electron microscope (ESEM, Carl Zeiss, EVO LS10, Oberkochen, Germany) with an acceleration voltage of 30 kV, 120 Pa pressure, and a backscattered-electron detector (NTS BSD). The images were obtained in grayscale and stored in TIFF format with a resolution of 2048 × 1536 pixels [6,22].

### 2.4. Image Analysis

The DIA was performed using the software ImageJ (v1.53k National Institutes of Health, Bethesda, MD, USA). The images obtained from SM were converted to 8-bit (grayscale) format and segmented using the threshold tool into three zones, namely, total, annular (mesocarp), and core (seeds and placental), of the bean slice; afterwards, the images were converted to binary format. The binary images were used to determine the following morphometric descriptors based on the projected area of the bean’s cross-section: area (A) for each of the three zones mentioned above, Feret’s diameter (FD) for the total and core area, and circularity of the total bean’s cross-section. In addition, the shrinkage ratio after each curing cycle based on the area (Ψ_A_) and Feret’s diameter (Ψ_FD_) were determined using Equations (1) and (2), based on equations proposed by Hernández et al. [23], Khraisheh et al. [24], Guiné et al. [25], Pinto and Tobinaga [26], and Tapia et al. [27].

(1) $$\psi_{A}=\frac{\left(A_{FV}-A\right)}{A_{FV}}$$

(2) $$\psi_{FD}=\frac{\left(FD_{FV}-FD\right)}{FD_{FV}}$$

where

A: projected area of the bean’s cross-section zone after each curing cycle.

A_FV_: projected area of the vanilla green bean’s cross-section zone.

FD: Feret’s diameter of the projected area of the bean’s cross-section zone after each curing cycle.

FD_FV_: Feret’s diameter of the projected area of the vanilla green bean’s cross-section zone.

Similarly, the evolution of the shrinkage ratio was determined within each killing technique, i.e., as compared to the area (Ψ_A_) and Feret’s diameter (Ψ_FD_) immediately after the killing technique was applied (C_0_), using Equations (3) and (4):

(3) $$\psi_{A}=\frac{\left(A_{C0}-A_{Cn}\right)}{A_{C0}}$$

(4) $$\psi_{FD}=\frac{\left(FD_{C0}-FD_{Cn}\right)}{FD_{C0}}$$

where

A_cn_: projected area of the bean’s cross-section zone for each curing cycle after scalding or HHP processing.

A_C0_: projected area of the bean’s cross-section zone immediately after (C_0_) scalding or HHP processing.

FD_Cn_: Feret’s diameter of the projected area of the bean’s cross-section zone for each curing cycle after scalding or HHP processing.

FD_C0_: Feret’s diameter of the bean’s cross-section zone immediately after (C_0_) scalding or HHP processing.

### 2.5. Statistical Analysis

The data are reported as average values and were subjected to variance analysis (ANOVA) using the Tukey difference test with level of significance α = 0.05 for the comparison of means (Minitab 20.3, Minitab Inc., State College, PA, USA, 2021).

## 3. Results and Discussion

### 3.1. Stereoscopic Observations

**Figure 1:**
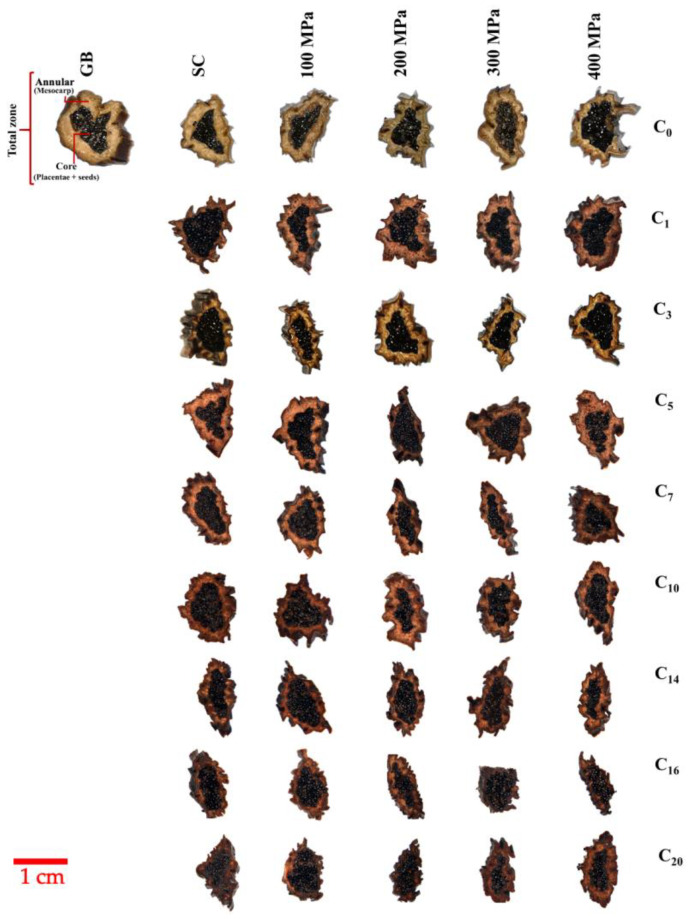
Stereoscopic observations of the HHP-assisted cured beans and scalded-cured beans. GB: green beans; Sc: scalding; C: curing cycle. Images show representative vanilla beans slices along the curing process, as observed from the top at 8× magnification and using reflected light for a bright field, after scalding and after applying the different HHP conditions studied.

Stereoscopic images of the different treatments of the vanilla beans are presented in Figure 1.

The results show that GB presents the structural parts of the total zone, annular (mesocarp), and core (seeds and placental) in a highly defined manner in comparison with the SC and HHP-treated beans. Through curing, the size of the annular zone was decreased. For SC, this effect was observed at C_1_, while at 100 and 300 MPa this effect was observed at C_3_. For the treatments at 200 MPa, the area of the annular zone was decreased at C_5_, whereas at 200 and 300 MPa, the decrease in annular area was observed from C_7_ to C_10_. However, at 400 MPa (C_7_), a higher annular zone was observed; this might be attributed to a thinner cell wall effect produced by the HHP treatment. At the end of curing (C_14_–C_20_), the area reduction of the annular zone was dependent on the curing cycle and not on the HHP-treatment, i.e., the cured beans showed this behavior under all pressure conditions. This trend suggests that the changes observed in morphology are primarily due to continuous changes in moisture content, rather than to the high pressure applied. In addition to pressure, the loss of water also contributed to the contraction of the sheath tissues. As found by Tapia [22], after C_10_, the moisture content decreased until reaching values below 20%; although the curing process is not a drying process as such, it intrinsically generates loss of water that induces the shrinkage of the tissue and the changes in microstructure. In general, it was possible to observe the elongation and compaction of vanilla beans during the HHP-assisted curing process, probably related to the moisture content variations in the beans.

### 3.2. Morphometric Parameters

**Figure 2:**
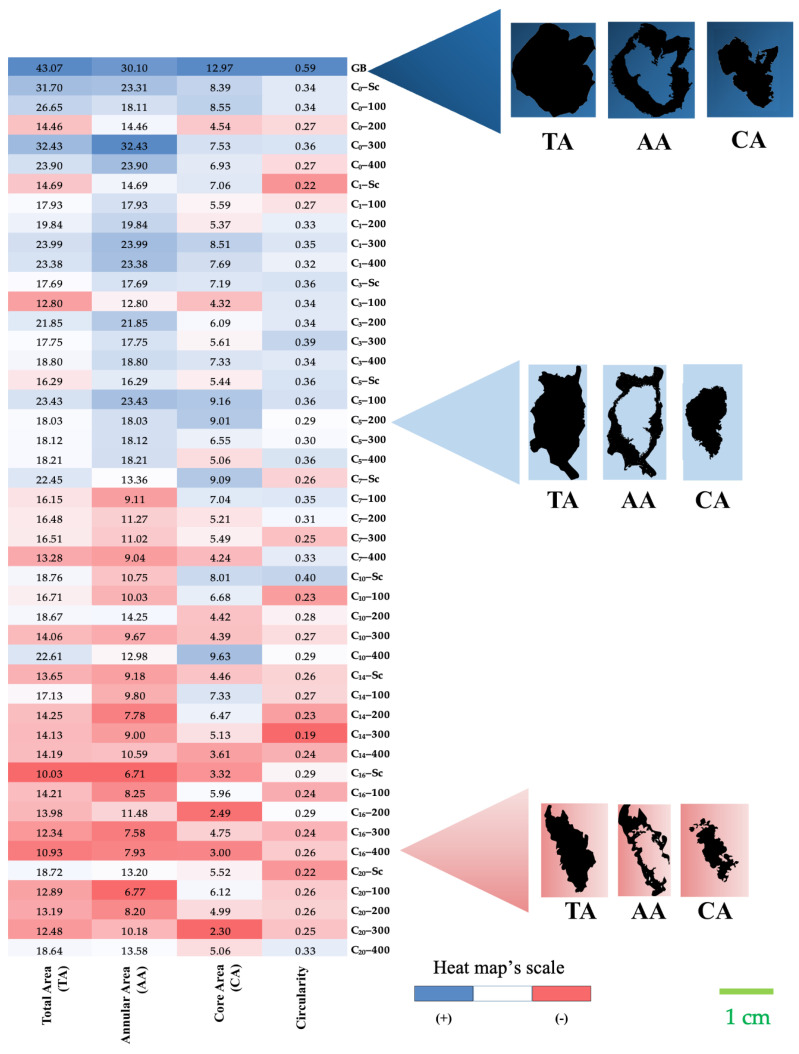
Heat map of mean values of the morphometric parameters determined during the curing of the HHP-treated and scalded vanilla beans: TA: total area, AA: annular area; CA: core area, and circularity. For comparison purposes, representative images of the digital image analysis applied to stereoscopic observations are presented for the green beans (GB) and HHP-treated vanilla beans under 200 MPa and 400 MPa. Sc: scalding; C: curing cycle. n = 4.

The DIA was carried out to determine the morphometric parameters of the total (TA), annular (AA), and core area (CA) of vanilla beans (Figure 2).

Green beans showed the highest average value of projected area in each segmented zone (TA = 43.07 mm^2^; AA = 30.10 mm^2^; CA = 12.97 mm^2^), whereas the minimum values of area were observed in HHP-treated beans at C_16_, after 400 MPa hydrostatic pressure processing (TA = 10.93 mm^2^; AA = 7.93 mm^2^; CA = 3.00 mm^2^) and after scalding (TA = 10.03 mm^2^; AA = 6.71 mm^2^; CA = 3.32 mm^2^). Although no statistically significant difference was found between these values, changes in the core area of the vanilla slices treated with HHP were observed under the stereomicroscope, so that the seeds inside the bean were no longer grouped only in the core zone, but also began to distribute along the bean zones and towards the epicarp. This change focused on the central area due to the use of HHP, mainly at 200 MPa and 400 MPa, can be explained by possible tissue disruption in comparison to non-pressure-treated vanilla beans; thus, changes in the core area of the beans due to the use of high pressures could be of potential advantage to promote the enzyme–substrate interactions.

In relation to circularity, values close to 1 indicate geometry relationships equivalent to those of a circular shape, i.e., circularity values considerably lower than 1 are characteristic of irregular elongated shape. The results of the image analysis indicate that this parameter decreased for all the treatments, resulting in cross-sections with increasingly elongated shape. The average circularity of green bean slices was 0.59, while as the conditioning cycles progressed the values oscillated between 0.19 and 0.39. Comparable circularity values (sometimes called as shape factor or shape parameter) for green vanilla pods were reported by Tapia et al. [22]. A similar effect on circularity value after HHP processing was observed in high-pressure-processed carrots by Trejo et al. [14], who reported a decrease in the value of this shape parameter in samples treated at 300 to 550 MPa (0.45–0.50), as compared to the value found in fresh carrots (0.75). Furthermore, in the case of vanilla beans, circularity values decreased along the sun-sweating cycles, regardless of the killing technique applied. This trend in circularity values suggests that the elongated shape would derive from differences in the physical opposition manifested by the tissues that constitute the bean microstructure against the applied pressure as the decrease in moisture content progressed.

### 3.3. Shrinkage Ratio

**Figure 3:**
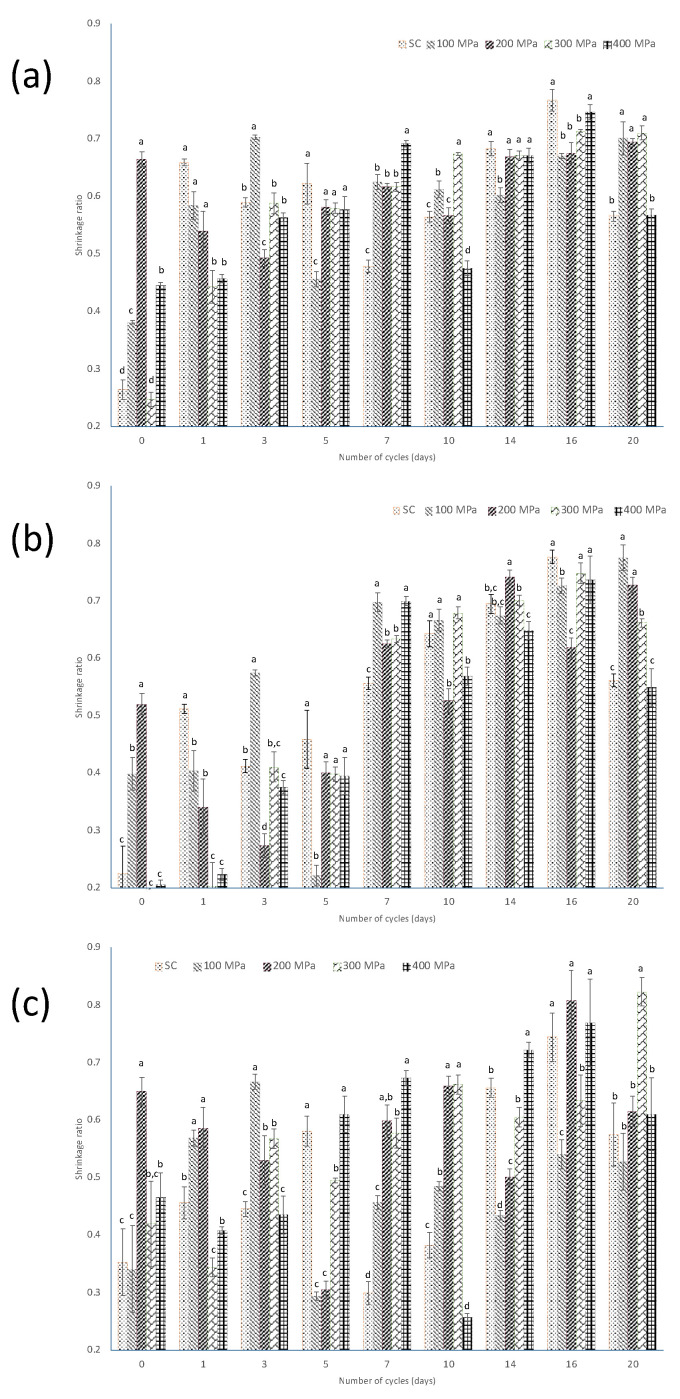
Evolution of the shrinkage ratio, as calculated by using Equation (1), of the (**a**) total area; (**b**) annular area; and (**c**) core area based on the cross-section’s projected area of the green beans, after the application of scalding (SC) and HHP at 100 MPa, 200 MPa, 300 MPa and 400 MPa along the curing process. Different small letters indicate statistically significant difference within each cycle.

Figure 3 shows the scalded and HHP-treated samples for the different curing cycles, based on the total, annular, and central areas with respect to the green beans.

The results in Figure 3a show that the most pronounced shrinkage was found at C_16_ after the scalding treatment, as well as after 300 and 400 MPa HHP treatment. In the annular area (Figure 3b), it was observed that from C_7_ onwards important shrinkage occurred, except at C_10_ for 200 MPa treatment, where the shrinkage value was between 0.4 and 0.6. This is consistent with results reported by Tapia et al. [19]; after ten sun-sweating cycles, deformation of the cells of cured vanilla beans was observed, and they also found a pronounced shrinkage in the mesocarp area. In the present work, in the case of the core area (Figure 3c), the most pronounced shrinkage was observed in C_16_ with scalding, 200, and 400 MPa and C_20_ at 300 MPa. The decrease in the central area may be due to cell damage caused by HHP, as reported by Yi et al. [13], who observed alterations in cell morphology in asparagus treated with HPP, especially in those treated at 400 and 600 MPa, finding extensive disruption and disorganization of cellular structures.

**Figure 4:**
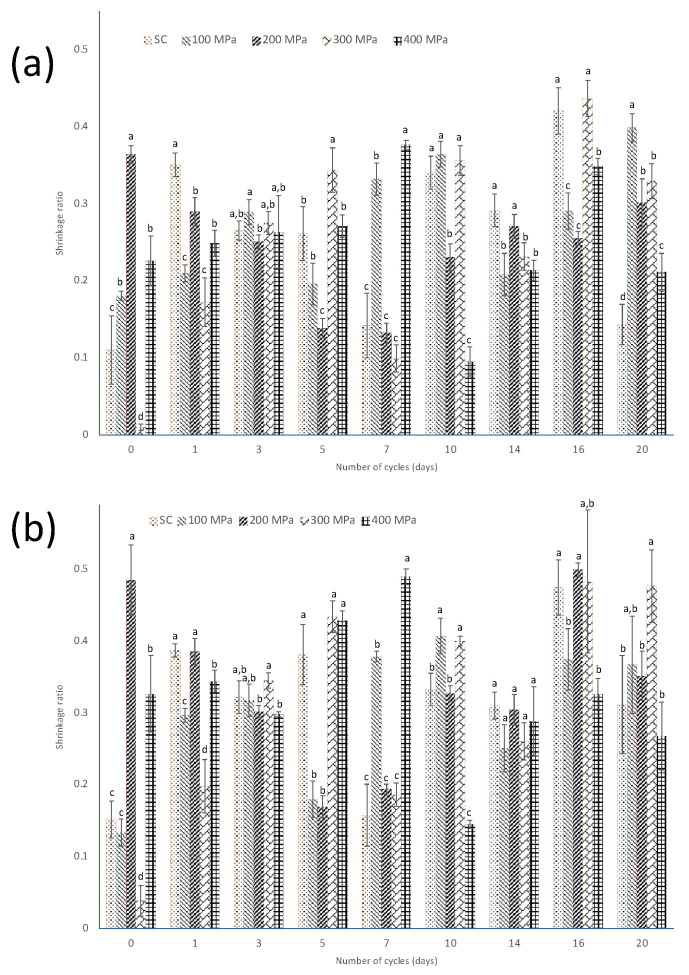
Evolution of the shrinkage ratio, as calculated by using Equation (2), of the (**a**) total area and (**b**) core area based on Feret’s diameter of the cross-section’s projected area of the green beans, after the application of scalding (SC) and HHP at 100 MPa, 200 MPa, 300 MPa, and 400 MPa along the curing process. Different small letters indicate statistically significant differences within each cycle.

As shown in Figure 4a, the smallest decrease in Feret’s diameter in total area was treatment, and the greatest shrinkage ratio based on this morphometric parameter was observed. Therefore, it was noticeable that the behavior of the shrinkage ratio based on this parameter is due to the decrease in moisture content during the sweating–drying cycles. With respect to the FD of the core area (Figure 4b), a greater shrinkage was observed at C_16_ after both killing techniques, scalding and HHP under 200 and 300 MPa; in addition, at C_0_ after 200 MPa, the beans showed a pronounced shrinkage.

**Figure 5:**
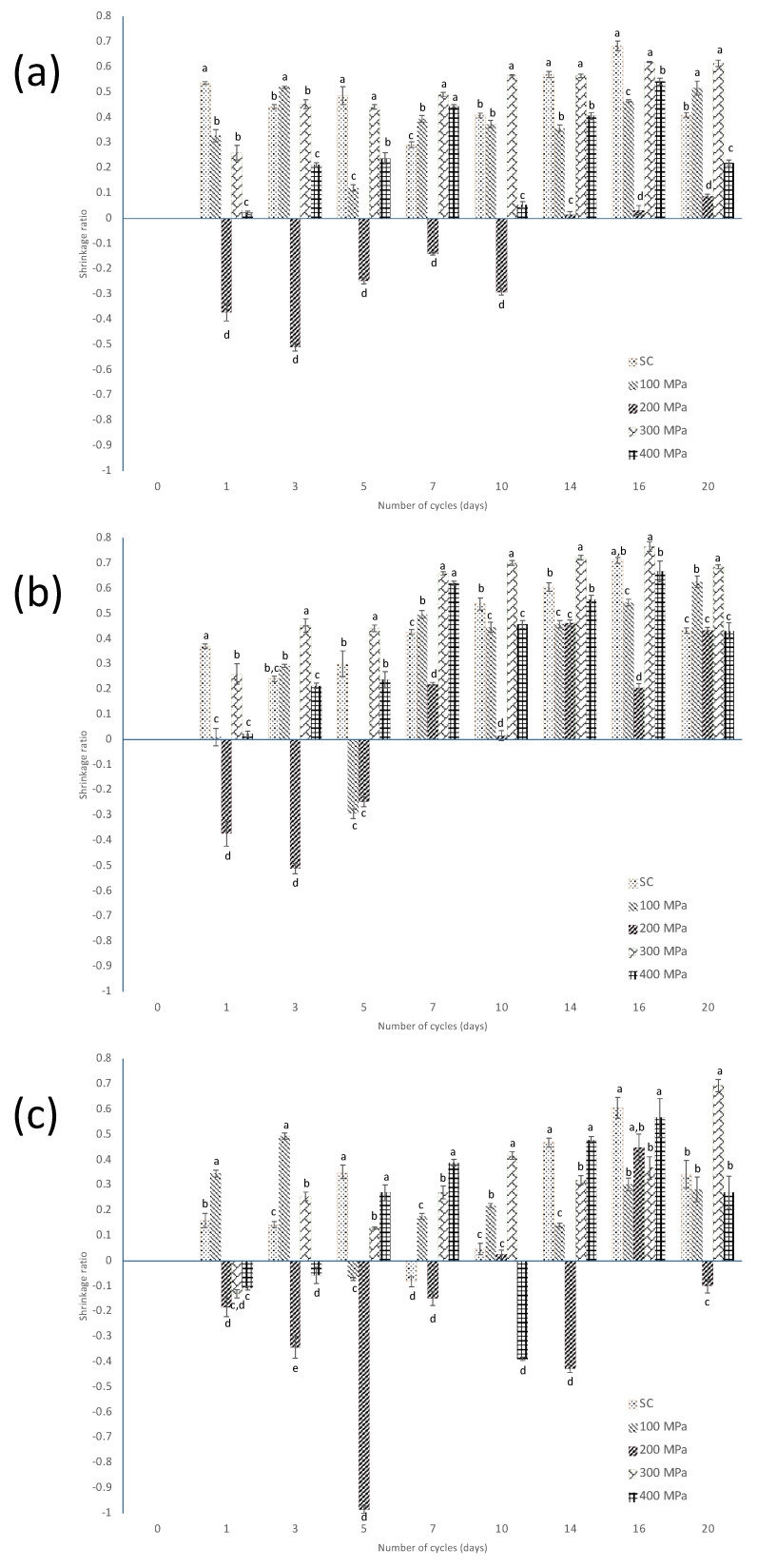
Evolution of the shrinkage ratio, as calculated by using Equation (3), of the (**a**) total area; (**b**) annular area; and (**c**) core area based on the cross-section’s projected area of the corresponding beans in cycle 0, after the application of scalding (SC) and HHP at 100 MPa, 200 MPa, 300 MPa, and 400 MPa along the curing process. Different small letters indicate statistically significant differences within each cycle.

Figure 5 shows the evolution of the shrinkage ratio of the area for the different zones analyzed, with respect to the area measured at C_0_. For 200 MPa treatment, noticeably, an enlargement of the area was observed during the first cycles in the total and annular regions, while the core area doubles its size at C_5_. At C_20_, no shrinkage was observed. Zhixuan et al. [28] mention that the use of HPP maintains better the texture of pickled kohlrabi, compared to heat treatment, which is because of more uniform moisture distribution; hence, it could be inferred that the increasing area of the cross-section of beans treated with HHP may be due to a better distribution of moisture content throughout curing.

**Figure 6:**
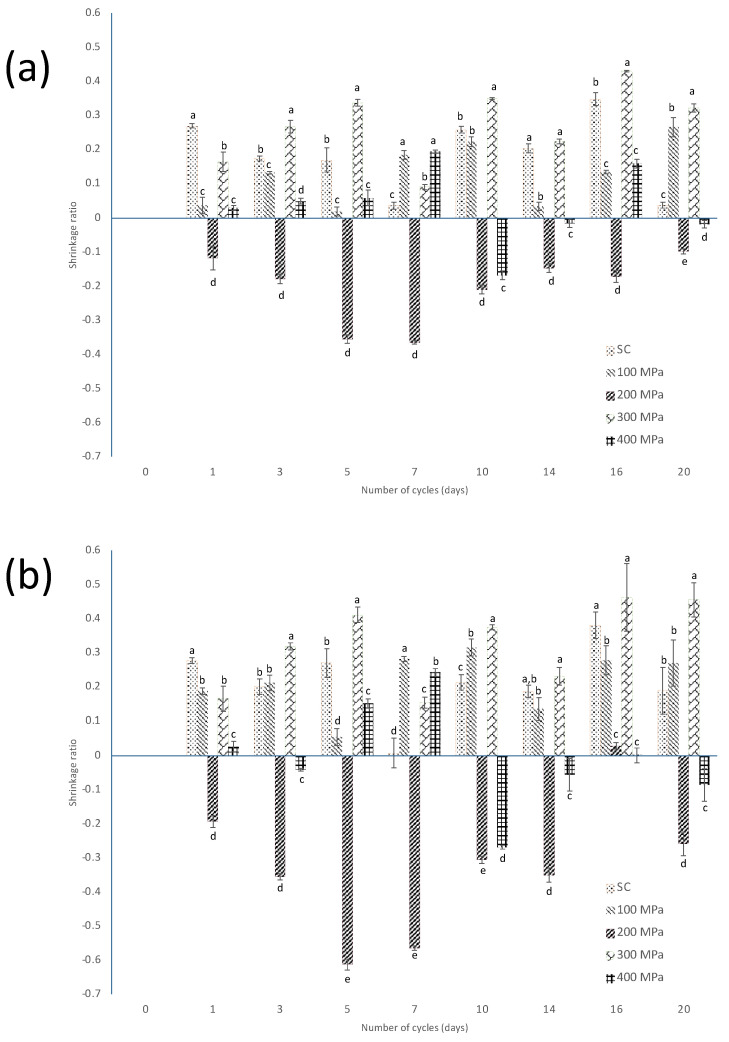
Evolution of the shrinkage ratio, as calculated by using Equation (4), of the (**a**) total area and (**b**) core area based on Feret’s diameter of the cross-section’s projected area of the corresponding beans in cycle 0, after the application of scalding (SC) and HHP at 100 MPa, 200 MPa, 300 MPa, and 400 MPa along the curing process. Different small letters indicate statistically significant differences within each cycle.

In relation to shrinkage ratio with respect to the FD measured at C_0_ (Figure 6), a similar trend was found after 200 MPa and 400 MPa processing, i.e., the FD of the bean slices’ projected area was greater as the sweating–drying cycles progressed. Thus, certain HHP conditions showed an increase in the cross-section’s area, probably related to moisture content.

### 3.4. Observations under CLSM

**Figure 7:**
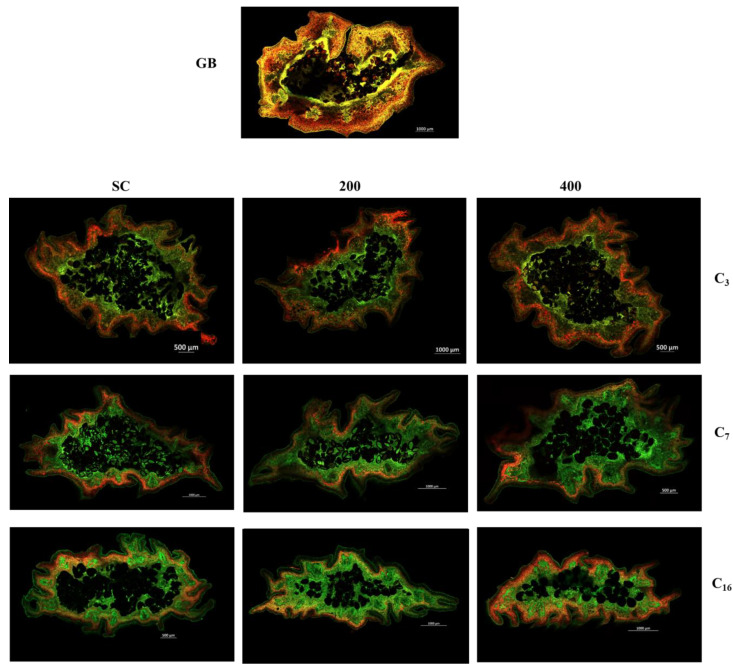
CLSM images of the bean’s cross section of green vanilla beans (GB) and vanilla beans after scalding (SC) and HHP treatments at 200 and 400 MPa (columns 200 and 400, respectively) in cycles 0, 3, and 16 (C3, C7, and C16, respectively). Two distinctive spectra (reddish and greenish colors), which also were distributed in different regions of the vanilla bean’s cross-section, were identified.

In Figure 7, autofluorescent zones related with the presence of different compounds in vanilla beans are shown. In GB, it was possible to observe orange and yellow spots whereas the green and red autofluorescence spectra were found in all areas of the bean slices. As the curing cycles progressed, the red spectrum was mainly present in the epicarp area, while decreasing in intensity as it approached the core area. On the other hand, green autofluorescence was mainly present in the core of the beans and spread to the epicarp; this might be attributed to the presence and dispersion of phenolic compounds, since these compounds are found throughout the curing cycle. Given that cell walls and membranes are structures composed of lipids, phospholipids, proteins, and lignocellulosic compounds, they also could exhibit autofluorescence when excited under green to ultraviolet wavelengths (for instance, 488 nm, 540 nm, and 630 nm) [29].

### 3.5. ESEM Analysis

**Figure 8:**
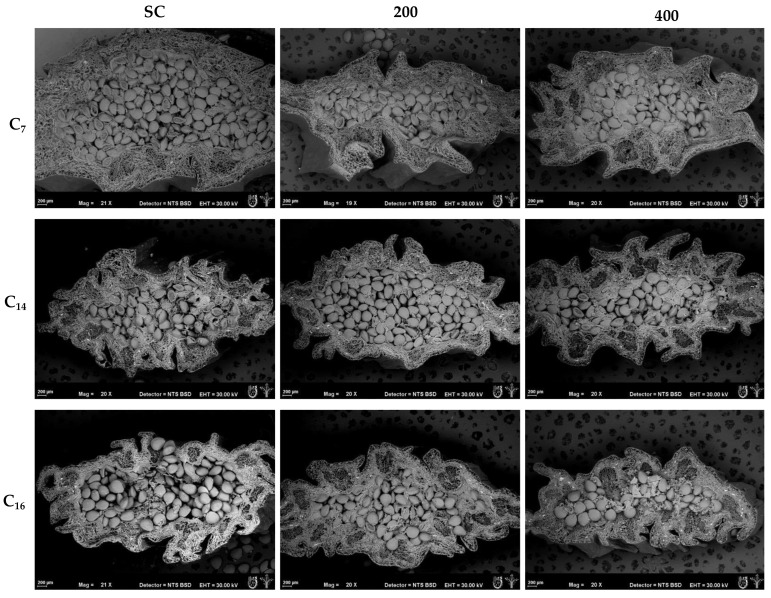
ESEM images of the bean cross sections after scalding (SC) and HHP treatments at 200 and 400 MPa (columns 200 and 400, respectively) in cycles 7, 14, and 16 (C_7_, C_14_, and C_16_, respectively).

The decrease in the projected area of the annular zone during the treatment under HHP at C_7_, as compared to scalding, was clearly observed through ESEM. Alterations in cell morphology as well as displacement of cellular tissues were observed as the curing cycles progressed (Figure 8). Amorphous white particles suggest the presence of damaged cells, mainly found after 400 MPa HHP; this agrees with Trejo et al. [10], who reported that during pressure treatments, morphometric changes take place, including changes in cell morphology, cell elongation, separation, or presence of cell fissures and/or cell wall disruption. For the case of amorphous particles, Yi et al. [9] found this type of cell disruption when treating asparagus at 400 and 600 MPa for 5 min. Additionally, Préstamo et al. [15] and Vázquez-Gutiérrez et al. [16] observed important structural changes in cauliflower and spinach, the former, and in persimmon, the latter, after using HHP at conditions slightly higher than those studied in the present work. This seems to indicate that a threshold in structural integrity could be exceeded around 400 MPa; therefore, in the case of vanillin beans, HHP at 200 MPa might be recommended as a combination useful for killing in vanilla curing, given the notorious presence of fluorescent compounds (observed via CSLM) and the decrease in shrinkage ratio found along the curing cycles.

## 4. Conclusions

The decrease in total area did not depend on the killing technique applied but instead on the curing cycle, as the set of microscopy observations allowed us to see. An image analysis showed that the core area of the beans was affected by HHP treatment since C_0_, while modifications in this zone after scalding were not significant until advanced cycles (C_14_). In the case of the annular zone, HHP at 400 MPa caused the highest decrease in area at C_7_, attributed to cell disruption. The autofluorescence observed via CSLM suggests some compounds that tend to concentrate in the epicarp, whereas other compounds tend to concentrate in the annular zone regardless of the killing technique used. In addition, cell disruption and cross-section shrinkage ratio are influenced mainly by the curing cycles and the subsequent decrease in moisture content. Despite the morphometric parameter used, or the zone of the vanilla bean cross-section analyzed, the shrinkage ratio with respect to either parameter measured just after applying the studied killing technique (cycle 0) disclosed the consistently different behavior generated by HHP under 200 MPa, which seems to be a useful HPP condition for curing vanilla beans. Therefore, we recommend a deeper exploration on the effects caused to vanilla bean microstructure and aroma profile when this combination of HHP operating conditions is applied.

## Data Availability

Data is contained within the article.
